# Sleep and breathing in premature infants at 6 months post-natal age

**DOI:** 10.1186/s12887-014-0303-6

**Published:** 2014-12-16

**Authors:** Yu-Shu Huang, Teresa Paiva, Jen-Fu Hsu, Ming-Chun Kuo, Christian Guilleminault

**Affiliations:** Sleep Center and Child Psychiatry Department, Chang Gung Memorial Hospital and Chang Gung University, Taoyuan, Taiwan; Clinical Neurosciences Department, Faculdade Medicina Lisboa, University of Lisbon, Lisbon, Portugal; Division of Pediatric Neonatology, Department of Pediatrics, Chang Gung Memorial Hospital and Chang Gung University, Taoyuan, Taiwan; Stanford University Sleep Medicine Division, 450 Broadway Street, MC 5704, Redwood City, CA 94063 Stanford USA

**Keywords:** Sleep questionnaire, Sleep-disordered breathing, Prematurity, Full-term infant

## Abstract

**Background:**

Poor sleep contributes to the developmental problems seen in preterm infants. We evaluated sleep problems in preterm infants 6 months of post-gestational age using the subjective Brief Infant Sleep Questionnaire (BISQ) and objective sleep tests. We also compared the sleep of premature infants with that of full-term infants.

**Methods:**

The study included 68 6-month-old full-term healthy infants and 191 premature infants born at <37 weeks gestation. All parents completed the BISQ-Chinese version and sleep diaries. At the same time, all premature infants were submitted to one night of polysomnography (PSG) in the sleep laboratory and also were set up with an actigraph kept for 7 days. Statistical analyses were performed using correlation coefficients and the t-test with SPSS version 18 to compare questionnaire responses with other subjective and objective measures of sleep.

**Results:**

The sleep problems indicated in the subjective questionnaire for the premature infants, particularly: “the nocturnal sleep duration, number of night awakenings, daytime sleep duration, duration of time with mouth breathing, and loud-noisy breathing” had significant correlations with sleep diaries, actigraphy and PSG results. The BISQ showed that duration of infant’s sleeping on one side, nocturnal sleep duration, being held to fall asleep, number of nighttime awakenings, daytime sleep duration, subjective consideration of sleep problems, loud-noisy breathing, and duration spent crying during the night were significantly different between the premature infants and the term infants. PSG confirmed the presence of a very high percentage (80.6%) of premature infants with AHI > 1 event/hour as indicated by the questionnaire.

**Conclusion:**

Premature infants have more sleep problems than full-term infants, including the known risk of abnormal breathing during sleep, which has been well demonstrated already with the BISQ-Chinese (CBISQ).

**Electronic supplementary material:**

The online version of this article (doi:10.1186/s12887-014-0303-6) contains supplementary material, which is available to authorized users.

## Background

Sleep is essential to human life and developmentally involves both physiologic and mental processes. During infancy, humans spend a majority of time in sleep [1-4]. Sleep is recognized not only as a resting state, but also as a state of intense brain development during which neurotransmitters specific for each sleep stage impact brain maturation [2,5-7].

Numerous studies have shown differences in sleep quality between premature and full-term infants [3-6,8-12]. Premature infants often have many problems during sleep, and the more premature they are the more problems are seen [6,8]. However, when sent home, the infant is considered by the pediatrician to have passed any life-threatening situations. Also, it seems that premature infants develop sleep-wake cycles differently from full-term infants, but that maturation of sleep is related to both time since birth and gestational age [9,12]. Many maturational events occur during sleep. Some parental reports indicated that 21% of children born prematurely are habitual snorers [13-15]. Premature infants have the well-known risk of abnormal control of breathing during sleep just after birth, and sleep apnea of prematurity has been well described [13-15]. Their presence usually leads premature infants to stay in the hospital setting till judged to have normal control of breathing during sleep. Many functions are altered in premature infants, and more so the more premature the infant is. Often there is generalized hypotonia, well known by specific neurological exams including regular follow-up of the presence of the “scarf-sign” [16], but there is also immaturity of many functions, including poor sucking and poor swallowing, and often lack of coordination of sucking, swallowing and breathing, leading to important and well documented oxygen saturation drops during feeding when such dysfunctions exist [17,18]. The sleep breathing problems also may be due to a lack of tone in the upper airway followed by collapse and obstruction or diaphragmatic movement dysfunction with immaturity of reflexes. Therefore, sleep-disordered breathing (SDB) is often noted in premature infants [14,15]. In the recent past, we made the preliminary observation that premature infants may have oral-facial developmental growth problems [18]. Such difficulties may lead to specific breathing problems during sleep and impair the sleep of the infants.

Our study focuses on the sleep of premature infants at 6 months of post-natal age who were born between 24 and 36 weeks of gestational age but had been considered to be in sufficient good health to be sent home without any treatment, and who were having only regular post-natal visits scheduled with the pediatrician. We compared our findings to those simultaneously obtained in a small group of full-term infants born during the same period.

## Methods

### Subjects

During the years 2010–2012, women delivering before 37 weeks of gestational age were asked to enroll their neonates prior to discharge from the neonatal intensive care unit of Chang Gung Memorial Hospital, a medical center with a pediatric department in Taiwan as part of the preterm infant group. Both boys and girls were included. The full-term infant group, with a gestational age ranging from 37 to 40 weeks and birth body weight of more than 2500 grams, was enrolled from the neonatal outpatient clinic in our hospital.

#### Inclusion criteria

- All neonates born in our hospital before 37 completed gestational weeks, without presentation of exclusion criteria and with a signed parental consent form during the study period comprised the “preterm-infants group”.
- All neonates delivered at 37 to 40 weeks of gestational age with birth body weight of more than 2500 grams, without presentation of exclusion criteria and with a signed parental consent form during the study period comprised the “full-term-infants group”.

#### Exclusion criteria

- Neonates with severe physical impairments (such as severe congenital heart diseases, DiGeorge syndrome, congenital hydrocephalus, and kernicterus) due to perinatal insults or hypoxic ischemic encephalopathy. We also excluded bronchopulmonary dysplasia or still required oxygen support (nasal cannula) after discharge.
- Neonates with confirmed severe congenital malformations.

### Methods

1. After obtaining written parental informed consent to take part in an institutionally approved human subject protocol (Chang Gung Memorial Hospital IRB: 98-2670C), the infants meeting the inclusion criteria began participation in our prospective growth study during the first year of life.
2. Infants had a pediatric evaluation at a regular 6-month post-natal visit and a sleep clinical evaluation. As part of this sleep evaluation, all premature infants underwent one night of PSG (Embla N7000 PSG recording-sleep- system) in the pediatric sleep laboratory. The following variables were monitored: electroencephalography, electromyography, electrocardiography and electro-oculography. Respiration was monitored with a nasal-cannula-pressure transducer, oral thermistor, thoracic and abdominal inductive plethysmography bands and pulse-oximetry. The infants were continuously video-monitored. For PSG scoring, the recommendations of the AASM-2007 were followed [7].
3. All infants were set up with an actigraph (Philips Respironics actiwatch 2, with a small size well-suited for use with younger subjects or those sensitive to wrist-worn devices.) on the left leg of the non-dominant side. The equipment measured body movements and light exposure. It was placed on the infant at the time of the visit and kept for 7 days, and was analyzed with commercially available software with one point every 2 minutes and indicated activity/non-activity. The equipment was correlated with a log simultaneously kept by care-givers.
4. At the same time, the parents were asked to fill out a validated questionnaire, the “Brief Infant Sleep Questionnaire-Chinese version”, (CBISQ), at the regular 6-month and 7-month visits to check the consistency of responses. The CBISQ is derived from the Brief Infant Sleep Questionnaire (BISQ) [19] of Avi Sadeh Additional file 1. The original BISQ had 13 items distributed in 3 categories, evaluating sleep duration, night awakenings, and method of falling asleep of infants aged 29 months or younger. To validate the Chinese version of the BISQ, we first translated and then back-translated the BISQ from English to Chinese. The test–retest reliability of the CBISQ was acceptable. There was significant correlation between the repeated sleep measures for location of sleep (r = 0.678*), preferred body position (r = 0.796*), nocturnal sleep duration (r = 0.534*), method of falling asleep (r = 0.848*), difficulty in falling asleep (r = 0.785*), number of night awakenings (r = 0.439*), daytime sleep duration (r = 0.455*), and subjective consideration of sleep problems (r = 0.663*). In order to evaluate respiratory sleep problems compared with actigraphy and PSG, we added 3 questions to the BISQ to create the CBSIQ version; these included “time spent with mouth breathing” (r = 0.568*), “severity of loud-noisy breathing” (0.760*), and “time spent crying during the night” (r = 0.206).
5. The small group (n = 68) of full-term infants (the “controls”) whose parents signed the consent form underwent a similar evaluation and was studied the same way as the premature infants.

### Analysis

Pediatrician notes of the 6-month follow-up evaluation for both the premature and full-term infants were reviewed. Complaints with specific emphasis on sleep were collected. The responses to the CBSIQ were then tabulated. Responses of premature infants one month later to the same questionnaire were also tabulated, and actigraphy and PSG data were analyzed. The statistical software package SPSS, Version 18 was used for data analysis. Variables are presented as either mean ± standard deviation (SD) or frequency. We used the t-test and Chi-square test for evaluation of differences between these 2 groups. The Pearson correlation coefficient was used in analysis of correlation between questionnaire and sleep Lab data. The statistical significance was defined at the 0.05 level.

## Results

### Demographic data

We originally signed up 229 premature infants from the PICU, but only 191 (83.41%) completed the study when they were 6 months old. The 191 6-month-old premature infants in the preterm infants group comprised 99 boys and 92 girls with an average birth body weight of 1646.66 g. The average gestational age was 31.52 weeks, with a maximum of 36 weeks and minimum of 24 weeks. There were 68 6-month-old infants in the full-term infants group, including 34 boys and 34 girls with an average birth body weight of 3169.69 grams and average gestational age of 38.35 weeks (Table 1).

**Table 1:** **Demographic data of the study subjects**

		**Preterm infants (n = 191)**	**Full-term infants (n = 68)**	**P value**
Gender	Male	n = 99 (51.83%)	n = 34 (50%)	
	Female	n = 92 (48.17%)	n = 34 (50%)	
Birth body weight (g)		1646.66 ± 588.60	3169.69 ± 410.58	<0.001*
Birth body height (cm)		39.96 ± 5.11	49.61 ± 1.88	<0.001*
Head circumference (cm)		28.88 ± 3.33	34.25 ± 1.24	<0.001*
Gestational age (weeks)		31.52 ± 3.21	38.35 ± 1.43	<0.001*

Birth body weight, Birth body height and Head circumference were all assessed at birth.

### The subjective and objective sleep problems of the premature infants (Table 2)

**Table 2:** **Subjective and objective sleep problems of premature infants**

**Questionnaire**	**Objective sleep measure**	**r**	**P value**
**CBISQ**	**Criterion of sleep diary**	0.757	0.011*
Nocturnal sleep duration	Nocturnal sleep duration		
Number of night awakenings	Nocturnal awake time	0.632	0.002*
	Numbers of night awakening	0.580	0.001*
	Nocturnal sleep efficiency	−0.644	<0.001*
Daytime sleep duration	Daytime sleep duration	0.630	0.002*
Loud-noisy breathing	Daytime sleep duration	0.348	0.064^**+**^
**CBISQ**	**Actigraphy criterion**		
Time spent with mouth breathing	Awakening time during sleep	0.514	0.042*
	Nocturnal sleep efficiency	−0.255	0.013*
**CBISQ**	**PSG criteria**		
Nocturnal sleep duration	Nocturnal sleep duration	0.545	0.002*
Number of night awakenings	Desaturation index	0.636	0.003*
Daytime sleep duration	AHI in sleep	0.767	0.075^**+**^
	Awakening after sleep onset	0.350	0.080^**+**^
Time spent with mouth breathing	Obstructive apnea count	0.509	0.026*
Loud-noisy breathing	Arousal count	0.401	0.089^**+**^
	Obstructive apnea count	0.535	0.018*

*P value < 0.05. ^+^P value < 0.10.

AHI: Apnea-Hypopnea Index (events/hour); Desaturation index: desaturation events/hour; “Arousal count” means total number of arousal during sleep; “Obstructive apnea count” means total number of obstructive apnea during sleep.

We compared the subjective CBISQ to other objective data obtained from sleep diaries, actigraphy and PSG data. These items of CBISQ showed sleep measures of “nocturnal sleep duration, number of night awakenings, daytime sleep duration and loud-noisy breathing” were significantly correlated with sleep diary, actigraphy and PSG. Items such as “mouth breathing” and “loud-noisy breathing” correlated with “total number of obstructive apnea during sleep” in PSG. Longer “Daytime sleep duration” also correlated with AHI >1; We were interested in these correlations as “mouth breathing” and “Loud-noisy breathing” are common symptoms associated with pediatric obstructive sleep apnea. Moreover, higher AHI means severe OSA will interrupt sleep and increase night awakenings and then induce daytime sleepiness. Therefore, the trend in the correlation between “AHI” and “number of obstructive apnea” in PSG and “Daytime sleep duration” in CBISQ were important and meaningful. The additional questions in the CBISQ pertaining to respiratory sleep problems also were shown to correlate with PSG.

The PSG data showed that 80.6% of 6-month-old premature infants had an apnea–hypopnea index (AHI) >1 event/hour (mean AHI = 3.63 ± 3.24), mean SaO2 97.01 ± 1.00%, total sleep time 368.83 ± 55.05 mins, sleep efficiency 82.43 ± 14.66%, and REM 24.03 ± 6.43% at polysomnography.

### Comparison of the differences between full-term and premature infants (Table 3)

**Table 3:** **Comparison of the preterm-infants group and the full-term-infants group using the CBISQ**

**CBISQ Sleep measure**		**Preterm infants**	**Full-term infants**	**P value**
Location of sleep	Infant crib in a separate room	1.0%	0.0%	-
	Infant crib in parents’ room	33.0%	33.3%	0.965
	In parents’ bed	64.9%	66.6%	0.828
	Infant crib in room with sibling	1.0%	0.0%	-
Preferred body position	On his/her back	52.2%	51.0%	0.876
	On his/her side	37.7%	31.4%	0.397
	On his/her belly	10.1%	17.6%	0.193
Nocturnal sleep duration (minutes, mean ± SD)		544.87 ± 81.93	490.71 ± 134.48	0.027*
Nocturnal sleep-onset time^#^ (mean ± SD)		2.61 ± 1.31	2.53 ± 1.02	0.782
Method of falling asleep	While feeding	19.8%	23.1%	0.555
	Being rocked	23.4%	21.5%	0.752
	Being held	24.8%	23.1%	0.769
	In bed alone	12.2%	12.3%	0.986
	In bed near parent	19.8%	20.0%	0.969
Difficulty falling asleep^#^ (mean ± SD)		2.47 ± 1.93	2.16 ± 1.71	0.494
Number of night awakenings^#^ (mean ± SD)		2.28 ± 0.93	1.72 ± 0.67	0.014*
Daytime sleep duration (minutes, mean ± SD)		364.07 ± 152.1	271.67 ± 133.16	0.014*
Subjective consideration of sleep problems^#^ (mean ± SD)		1.53 ± 0.69	1.21 ± 0.54	0.024*
Time spent with mouth breathing^#^ (mean ± SD)		1.47 ± 0.57	1.20 ± 0.42	0.077^**+**^
Loud-noisy breathing^#^ (mean ± SD)		1.88 ± 0.69	1.44 ± 0.51	0.010*
Time spent with crying during night^#^ (mean ± SD)		1.64 ± 0.71	1.26 ± 0.45	0.003*

*P value < 0.05. ^+^P value < 0.10.

^#^These questions were evaluated by severity, and scored from 1 to 4.

Since our CBISQ was shown to be a reliable and valid tool for sleep measurement, we used the data to compare the differences in sleep between the preterm-infant and full-term-infant groups. The results revealed, as expected, a significant difference between the 2 groups: The premature group preferred a side body position and being held when falling asleep, had more night awakenings, greater subjective mention of presence of a sleep problem by caregivers, louder noisy breathing and more time spent crying during the night. Also, the premature infants had longer nocturnal and daytime sleep duration.

## Discussion

Based on the results described above, we consider the CBISQ a reliable and valid tool for the measurement of sleep problems in infants. It can be used to measure nocturnal sleep duration, number of night awakenings, daytime sleep duration, time spent breathing with the mouth and loud-noisy breathing during sleep. These measures may indicate the presence of underlying infancy sleep problems and sleep-disordered-breathing. Finally, to make the CBISQ an internationally available tool, it was submitted to a Chinese–English back-translation.

Not only did our study validate the CBISQ, but it is also the first study targeting the difference between premature and full-term infants through a reliable and valid screening questionnaire. Based on our results, premature infants, rather than full-term infants, preferred a side body position and being held when falling asleep. Apart from this, premature infants had longer nocturnal sleep duration, more night awakenings, and longer daytime sleep duration. The caregivers of premature infants noted that their children had more sleep problems. A significant difference between preterm and full-term infants in nocturnal sleep duration and loud-noisy breathing was noted. The number of night awakenings and time spent with mouth-breathing also varied between the 2 groups. These questionnaire findings indicated a higher prevalence of sleep-breathing disorders [20-22], and correlated with those obtained with PSG that showed a very high percentage (80.6%) of premature infants with AHI greater than 1 event/hour. These disorders then impact nocturnal sleep time and quality.

As a pilot study in this field, our results provide new insights into the difference between premature and full-term infants with regard to sleep problems. During the first year of life, infancy-sleep is a rapid maturational process [23,24], so the settling time, daytime sleep duration and time spent crying during the night may vary widely between repeated questionnaires. Infancy sleep maturation may impact the maturation of the central nervous system, overall functioning, and future cognitive, motor, and temperament development [25].

There are some methodological limitations that should be noted when interpreting our findings. First, the sample sizes of the 2 groups in our study were very different, so some of the differences between the premature and full-term infants may not be apparent in the CBISQ sleep measurement. Second, 16.59% of our study group (premature infants) did not complete the CBISQ assessment and sleep examination when they were 6 months old, but our follow-up study had more than 70% of the initially signed-up children. Third, actigraphy provides only integrated sleep data, not detailed parameters of the sleep status, so the validity testing of the CBISQ sleep measure with actigraphy was not ideal. Fourth, it is difficult to perform PSG in premature infants because of uncooperative child, night feeding and size of premature infant with too small limbs and torso for some equipments. However our premature group was large, and actigraphy and PSG data were both obtained from this large number of premature infants. But our study monitored simultaneous objective and subjective data investigating sleep problems of the premature.

Using this reliable and validated tool, the CBISQ, longitudinal studies surveying premature infants using sleep measurement and mental development every 3 months from birth can be designed to address the issue of delayed development in premature infants and how this correlates with sleep-breathing disorders or poor nocturnal sleep quality. Use of the CBISQ may help clinicians follow the evolution of sleep problems overtime and schedule sleep studies when needed.

## Conclusion

Infancy sleep maturation may impact the maturation of the central nervous system, overall functioning, and future cognitive, motor, and temperament development. Premature infants have more sleep problems than full-term infants, including the known risk of abnormal breathing during sleep, which has been well demonstrated already with the BISQ-Chinese (CBISQ).The longitudinal studies surveying premature infants using sleep measurement and mental development every 3 months from birth can be designed to address the issue of delayed development in premature infants and how this correlates with sleep-breathing disorders or poor nocturnal sleep quality.

## Additional file

Additional file 1:
**Brief Infant Sleep Questionnaire-Chinese version (CBISQ).** The CBISQ is derived from the Brief Infant Sleep Questionnaire (BISQ) [19] of Avi Sadeh. We translated the BISQ from English to Chinese.

## Abbreviations

BISQ: Brief infant sleep questionnaire
CBISQ: Brief infant sleep questionnaire-Chinese version
PSG: Polysomnography
AHI: Apnea-Hypopnea Index
PICU: Premature intensive care unit
