# Supplementary material for: Sleep and breathing in premature infants at 6 months post-natal age
Source: BMC Pediatr. 2014 Dec 16;14:303. doi: 10.1186/s12887-014-0303-6 (PMC4272529; doi:10.1186/s12887-014-0303-6)
Supplement: Additional file 1: — Brief Infant Sleep Questionnaire-Chinese version (CBISQ). The CBISQ is derived from the Brief Infant Sleep Questionnaire (BISQ) [19] of Avi Sadeh. We translated the BISQ from English to Chinese. [file 12887_2014_303_MOESM1_ESM.pdf]

中文版嬰幼兒睡眠問卷(CBISQ)

填寫問卷人姓名:______________ 日期:____年____月____日
填寫問卷人關係: ☐父親 ☐母親 ☐祖父母 ☐其他
兒童姓名:____________病歷號:_____________ 生日: ____年____月____日
性別: ☐男 ☐女 家中排行: ☐老大 ☐中間 ☐老么

請單選一項最適合的敘述 (關於你的小孩過去一週的睡覺狀況)

1.睡眠環境:
☐睡在單獨房間的嬰兒床 ☐睡在跟父母同房間的嬰兒床
☐睡在父母的床上 ☐睡在跟兄弟姐妹同房間的嬰兒床
☐其他

2.你的小孩最常睡覺的姿勢:
☐趴睡 ☐側睡 ☐仰睡

3.你的小孩夜間睡眠時間是多少?(晚上七點到早上七點之間)
______小時 _____分鐘

4.你的小孩白天睡眠時間是多少?(早上七點到晚上七點之間)
______小時 _____分鐘

5.平均晚上醒來次數:______

6.你的小孩在夜間醒來的時間有多長?(晚上十點到早上六點間)
______小時 _____分鐘

7.晚上你需要花多久時間讓小孩入睡?
______小時 _____分鐘

8.什麼情況下你的小孩會入睡?
☐餵食時 ☐搖他 ☐抱著
☐單獨在床上 ☐有父母陪在床上

9.你的小孩晚上通常幾點睡?
_______時_______分

10.你認為你小孩的睡眠是個問題嗎?
☐非常嚴重 ☐一點點 ☐完全不是問題

11.你的小孩睡覺的時候是張開嘴巴睡覺嗎？
☐非常嚴重 ☐一點點 ☐完全沒有

12.你的小孩睡覺的時候呼吸聲很大嗎？
☐非常嚴重 ☐一點點 ☐完全沒有

13.你的小孩晚上睡覺常常哭鬧嗎？
☐非常嚴重 ☐一點點 ☐完全沒有
